# Investigating white matter functional network connectivity across the Alzheimer’s disease spectrum using resting-state fMRI

**DOI:** 10.3389/fnimg.2026.1796824

**Published:** 2026-04-23

**Authors:** Vaibhavi S. Itkyal, Theodore J. LaGrow, Kyle M. Jensen, Armin Iraji, Vince D. Calhoun

**Affiliations:** 1 Wallace H. Coulter Department of Biomedical Engineering, Georgia Institute of Technology and Emory University, Atlanta, GA, United States; 2 Tri-institutional Center for Translational Research in Neuroimaging and Data Science, Georgia State University, Georgia Institute of Technology, Emory University, Atlanta, GA, United States; 3 Department of Neuroscience, Emory University, Atlanta, GA, United States; 4 College of Computing, Georgia Institute of Technology, Atlanta, GA, United States; 5 College of Lifetime Learning, Georgia Institute of Technology, Atlanta, GA, United States; 6 Department of Electrical and Computer Engineering, Georgia Institute of Technology, Atlanta, GA, United States

**Keywords:** ADNI, Alzheimer’s disease, functional connectivity, independent component analysis, resting-state fMRI, white matter

## Abstract

White matter (WM) has traditionally been considered structurally important but functionally inert in fMRI research. However, growing evidence indicates that WM exhibits meaningful BOLD fluctuations and participates in functional connectivity. Here, we investigate alterations in WM functional network connectivity (FNC) across the Alzheimer’s disease (AD) spectrum using resting-state fMRI data from the Alzheimer’s Disease Neuroimaging Initiative (ADNI; 415 cognitively normal (CN), 283 mild cognitive impairment (MCI), 91 AD). We applied a guided independent component analysis (ICA) approach based on a combined multiscale template including 202 intrinsic connectivity networks [ICNs; 97 WM, 105 gray matter (GM)] to estimate subject-specific timecourses and compute FNC. Group differences in WM–WM, GM–GM, and WM–GM functional network connectivity (AD–CN, AD–MCI, MCI–CN) were evaluated using two-sample t-tests on residual FNC values for age, sex, and mean framewise displacement. Multiple comparisons across edges were controlled using false discovery rate correction (q < 0.05), and effect sizes were quantified using Hedges’ g. Results showed robust alterations in WM–WM and WM–GM connectivity in AD, particularly involving WM subcortical, frontal, sensorimotor, and occipitotemporal networks. Several WM–GM interactions with cerebellar and hippocampal GM networks were also disrupted, including reduced GM–cerebellar: WM–frontal coupling and increased GM–hippocampal: WM–frontal connectivity. Notably, MCI already showed WM–GM dysconnectivity relative to CN, suggesting that functional disruption of WM circuits emerges prior to overt dementia. These findings provide converging evidence that WM functional connectivity is both measurable and selectively altered across the AD continuum. Our findings support WM FNC as a candidate biomarker to GM-based measures for staging and monitoring AD. Together, these results position WM–GM dysconnectivity as an important systems-level signature of the AD continuum and support WM functional network connectivity as a promising complement to established GM-based measures for understanding disease progression.

## Introduction

Alzheimer’s disease (AD) is a progressive neurodegenerative disorder characterized by memory impairment, cognitive decline, and large-scale disruption of brain networks. While most functional MRI (fMRI) studies have focused on gray matter (GM) pathology and connectivity, converging evidence suggests that white matter (WM) also plays a critical role in AD progression (Bubb et al., 2018; Kaskikallio et al., 2019). Diffusion imaging studies consistently report microstructural abnormalities in WM, including myelin degradation and axonal loss, across the AD continuum (Mito et al., 2018). Yet, the functional contribution of WM to AD-related network changes remains comparatively underexplored.

Historically, WM has been largely neglected in fMRI analyses because of its lower blood-oxygenation level-dependent (BOLD) signal amplitude and concerns about vascular and partial-volume effects. Recent work, however, has shown that WM exhibits structured, low-frequency BOLD fluctuations that are temporally coherent, spatially organized, and reproducible across datasets and sites (Peer et al., 2017; Gore et al., 2019). These findings challenge the view of WM as functionally “silent” and instead support the idea that WM participates actively in large-scale communication between GM regions.

Independent component analysis (ICA) has been particularly useful for characterizing WM functional organization. ICA-derived intrinsic connectivity networks (ICNs) have been identified within WM that are distinct from GM networks and show stable patterns across individuals and cohorts (Itkyal et al., 2025). Such WM ICNs can be grouped into functional subdomains (e.g., sensorimotor, occipitotemporal, frontal, subcortical), providing a systems-level framework to study WM functional involvement in health and disease. In parallel, extensive work on GM ICNs has revealed consistent alterations in default mode, hippocampal, frontal, and sensorimotor networks in AD and mild cognitive impairment (MCI), the intermediate clinical stage of the disease (LaGrow et al., 2025a, 2025b). Recent work by Xu et al. (2024), using a seed-based approach to link WM tract seeds and GM ROIs, demonstrated that white matter exhibits meaningful functional changes across the AD spectrum, providing important evidence that WM is not functionally inert but actively altered in disease. The current study extends this line of investigation by employing a hybrid-data driven and comprehensive joint template approach that simultaneously models 97 WM and 105 GM intrinsic connectivity networks at scale (789 ADNI participants: 415 CN, 283 MCI, 91 AD), enabling systematic characterization of stage-specific within-tissue and cross-tissue connectivity alterations across the disease continuum.

Functional network connectivity (FNC) computed from ICN time course offers a compact summary of large-scale interactions among brain networks. FNC, obtained from pairwise correlations over the full scan, has been widely used to identify modular alterations in GM connectivity in AD. However, most existing studies focus exclusively on GM or treat WM as nuisance signal, leaving open the question of how WM functional connectivity and WM–GM interactions change across the AD continuum. A joint analysis of WM and GM FNC could provide a more complete picture of network degradation and reorganization in AD, and may reveal patterns that are not apparent from GM-only analyses.

In this brief report, we leverage a guided ICA framework that combines a large-scale, validated WM ICN template (97 networks freely available at: https://trendscenter.org/data/) with a GM network template (105 networks) (Iraji et al., 2023; Jensen et al., 2024) to estimate subject-specific ICNs and whole-brain FNC in a large Alzheimer’s Disease Neuroimaging Initiative (ADNI) cohort. Using resting-state fMRI data from cognitively normal (CN), MCI, and AD participants, we examine group differences in WM–WM, GM–GM, and WM–GM connectivity across the disease spectrum. We hypothesized that: (i) WM–WM and WM–GM FNC would show robust alterations in AD relative to CN, particularly in posterior and subcortical-frontal pathways implicated in AD; and (ii) WM-related dysconnectivity would be observed even at the MCI stage of disease progression, indicating that WM functional changes emerge prior to overt dementia. By explicitly incorporating WM into a network-level framework, this work aims to evaluate the potential of WM functional connectivity as a potential imaging biomarker for AD.

## Methods

The complete analysis pipeline, including fMRI preprocessing, template-guided ICA, extraction of WM and GM IC time courses, FNC computation, covariate regression, and group-level statistical testing with FDR correction, is summarized in Supplementary Figure 1.

### Participants

This study utilized resting-state functional magnetic resonance imaging (rs-fMRI) data from the ADNI (Mueller et al., 2005). The sample included 789 participants spanning the AD spectrum: 415 CN, 283 individuals with MCI, and 91 individuals with a clinical diagnosis of AD. Diagnostic classification was based on ADNI consensus criteria, incorporating clinical evaluation, cognitive testing, and informant reports. Participants were included if they had a usable rs-fMRI scan, corresponding high-resolution T1-weighted anatomical image, and complete demographic information. All data were acquired on 3 T MRI systems at ADNI sites using standardized protocols; detailed acquisition parameters are available in ADNI documentation and are summarized in the Supplement. Demographic characteristics for each diagnostic group (including age and sex distribution) are listed in Table 1. Subject inclusion criteria and clinical diagnoses followed ADNI protocols, and all procedures were approved by local institutional review boards with informed consent obtained from each participant.

**Table 1:** Group-wise summary statistics for CN, MCI, and AD participants.

Research group	# Scans (F/M)	Age, mean ± SD (years)
CN	415 (247/168)	71.20 ± 6.45
MCI	283 (126/157)	71.82 ± 7.38
Dementia	91 (45/46)	73.48 ± 7.80

#### fMRI preprocessing

All rs-fMRI data underwent standard preprocessing using a combination of SPM12 and FSL software [following the procedures outlined in the NeuroMark framework (Du et al., 2020)]. The first five volumes of each scan were discarded to allow for magnetization stabilization. The remaining volumes were then realigned using rigid-body motion correction to estimate and correct for head movement across the time series. Slice timing correction was applied to adjust for inter-slice acquisition delays relative to the middle slice.

Following motion and slice-timing correction, functional images were spatially normalized to the Montreal Neurological Institute (MNI) standard space using deformation fields estimated from each subject’s T1-weighted anatomical image. Normalized images were resampled to 3 mm^3^ isotropic voxels to provide a common resolution across participants. A Gaussian spatial smoothing kernel of 6 mm full-width at half maximum (FWHM) was then applied to improve signal-to-noise ratio while maintaining a balance between spatial specificity and sensitivity in both WM and GM. Itkyal et al. (2025) demonstrated that smoothing with template-guided ICA maintains tissue specificity without compromising WM signal integrity.

To ensure data quality and minimize motion-related confounds, we implemented stringent exclusion criteria at the subject level. Scans were discarded if they contained fewer than 120 usable time points, if the mean framewise displacement exceeded 0.25 mm, or if absolute head motion surpassed 3 mm of translation or 3 degrees of rotation in any direction. After applying these quality control thresholds, more than 700 scans across the three diagnostic groups (CN, MCI, AD) were retained for subsequent functional network and connectivity analyses.

#### WM and GM functional network templates and guided ICA

To characterize brain-wide functional organization, we utilized a previously validated WM ICNs template comprising 97 WM components. The WM ICNs were derived from a large-scale spatial ICA decomposition of individuals (Iraji et al., 2023) and subsequently selected and validated as a 97-component WM template (Itkyal et al., 2025). These WM ICNs were selected based on their spatial specificity, reproducibility, and low overlap with GM and CSF. The WM ICNs were grouped into functional subdomains (e.g., sensorimotor, occipitotemporal, frontal, subcortical, parietal), providing a systems-level representation of WM functional architecture. Details of the template derivation and subdomain labels are provided in prior work (Itkyal et al., 2025) and summarized in the Supplement. The WM ICN classification was performed using a multi-step approach combining automated spatial overlap analysis with the JHU WM atlas and AAL GM atlas, stability thresholds (>0.8 across model orders), and manual expert validation to ensure spatial specificity and low overlap with GM/CSF (Itkyal et al., 2025).

To model GM functional organization in parallel, we incorporated 105 ICNs from an established GM functional network template (Iraji et al., 2023; Jensen et al., 2024). These GM components include canonical resting-state networks such as default mode, hippocampal/medial temporal, sensorimotor, visual, cerebellar, and fronto-parietal systems. Together, the WM (97) and GM (105) templates yielded a combined set of 202 spatial priors that span both tissue types.

Subject-specific ICNs and their associated timecourses were estimated from each subject using a multi-objective optimization ICA with reference (MOO-ICAR) framework.^1^ This guided ICA approach imposes soft spatial constraints based on the template ICNs while capturing individual variability. In brief, for each subject, the algorithm optimizes a cost function that simultaneously maximizes statistical independence of components and spatial similarity to the WM and GM template maps. This yields subject-level ICN spatial maps that closely align with the group priors, along with corresponding timecourses that reflect the subject’s spontaneous BOLD fluctuations in each network. The WM ICN templates used as spatial priors were derived with explicit selection for spatial specificity and low overlap with GM and CSF, providing functional purity without requiring hard anatomical masking during subject-level decomposition.

By applying MOO-ICAR with the combined WM and GM template (using GIFT toolbox: https://trendscenter.org/software/gift/), we obtained 202 subject-specific ICNs per participant (97 WM, 105 GM), each with a single representative timecourse. These timecourses formed the basis for subsequent functional connectivity analyses of WM–WM, GM–GM, and WM–GM interactions.

**Figure 1:**
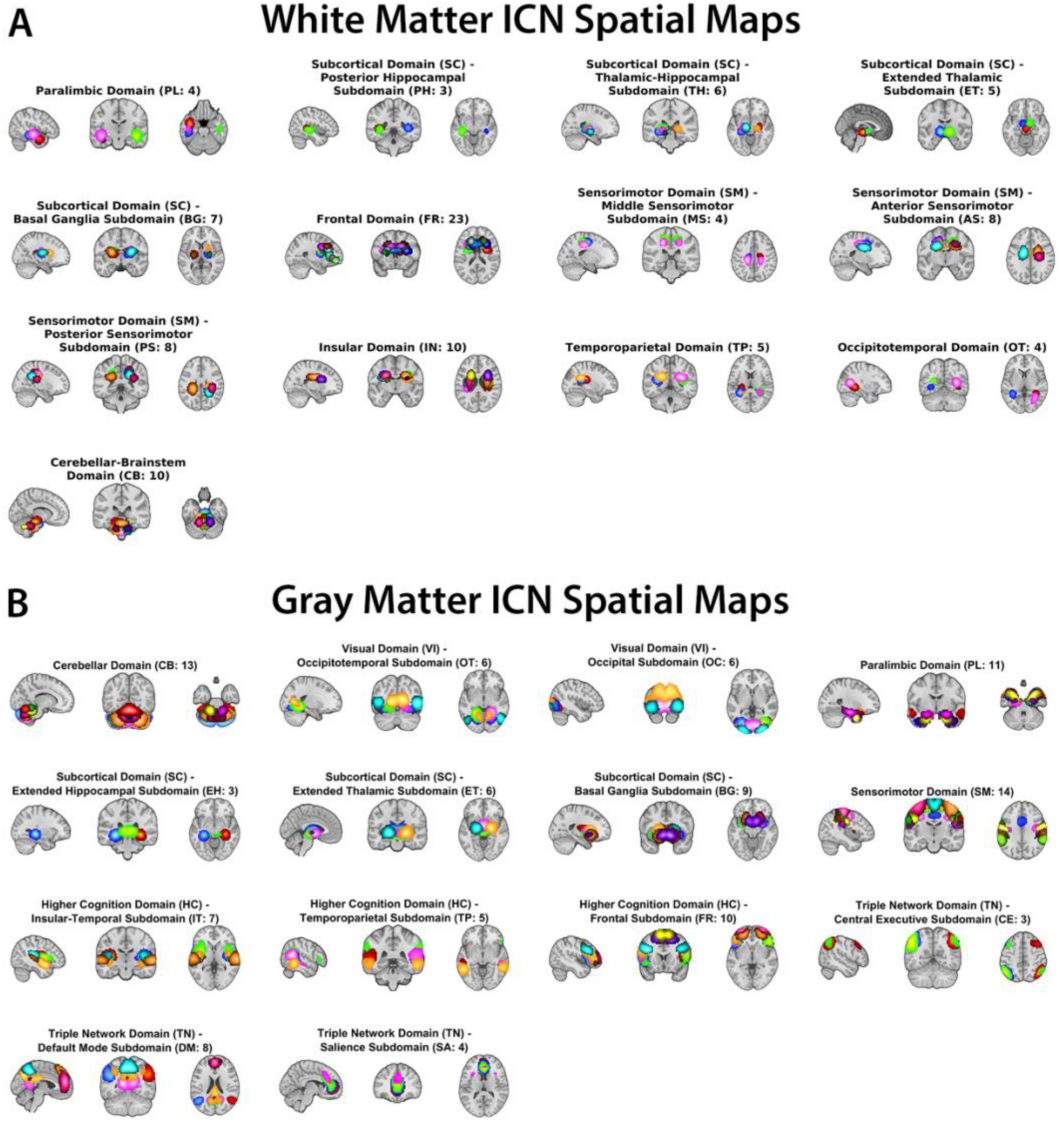
White matter **(A)** and gray matter **(B)** intrinsic connectivity network (ICN) templates.

Spatial maps of the (A) 97 WM ICNs and (B) 105 GM ICNs used as priors in the guided ICA framework (see Figure 1). ICNs were assigned to functional domains and subdomains based on spatial overlap with the JHU WM atlas and AAL GM atlas, cross-referenced with nearest GM regions, and reorganized using FNC patterns to group WM ICNs with functionally similar GM domains (Itkyal et al., 2025). WM ICNs are grouped into 13 functional subdomains (e.g., sensorimotor, occipitotemporal, frontal, subcortical, parietal), and GM ICNs are grouped into 14 canonical resting-state subdomains (e.g., default mode, hippocampal/medial temporal, sensorimotor, visual, cerebellar, fronto-parietal). ICN maps are displayed in MNI space and thresholded for visualization. These templates provide the spatial priors for estimating subject-specific ICNs spanning both WM and GM.

#### Functional connectivity and statistical analysis

FNC was computed from the ICN timecourses for each subject. Specifically, we calculated pairwise Pearson correlations between all 202 ICN timecourses (97 WM + 105 GM), yielding a 202 × 202 symmetric connectivity matrix per participant. Correlation coefficients were transformed to Fisher’s z-scores to improve normality, producing whole-brain FNC profiles for each individual.

Group-level functional network connectivity (FNC) edges were categorized based on the tissue type of the participating intrinsic connectivity networks (ICNs): (i) WM–WM, representing connections between two white matter ICNs; (ii) GM–GM, representing connections between two gray matter ICNs; and (iii) WM–GM, representing cross-tissue connections between a white matter and a gray matter ICN.

To account for potential confounding effects, age, sex, and mean framewise displacement were regressed from each subject’s FNC values using a general linear model (GLM) implemented in MATLAB (fitglm). Residuals from this model were retained as covariate-adjusted connectivity estimates for subsequent analyses.

Group-level mean FNC matrices were then computed separately for cognitively normal (CN), mild cognitive impairment (MCI), and Alzheimer’s disease (AD) groups to visualize overall network organization and qualitative differences across the disease spectrum. Group differences in connectivity were assessed on an edge-wise basis using two-sample (independent) t-tests (two-tailed), as implemented in MATLAB (ttest2), for three pairwise contrasts: AD vs. CN, AD vs. MCI, and MCI vs. CN. These tests were performed on the covariate-adjusted residual FNC values. Resulting *p*-values were corrected for multiple comparisons using false discovery rate (FDR) correction at q < 0.05 across all edges within each contrast.

Effect sizes were quantified using Hedges’ g, providing a bias-corrected standardized estimate of the magnitude of connectivity differences between groups. For visualization, FNC difference matrices display Hedges’ g values. The matrices are symmetric: the upper triangle shows FDR-thresholded results (non-significant edges, q > 0.05, are masked in gray), while the lower triangle displays the full, unthresholded effect size distribution for all edges. This approach enables simultaneous visualization of statistically significant effects and the complete distribution of effect sizes.

## Results

### Preserved large-scale WM and GM network organization across groups

**Figure 2:**
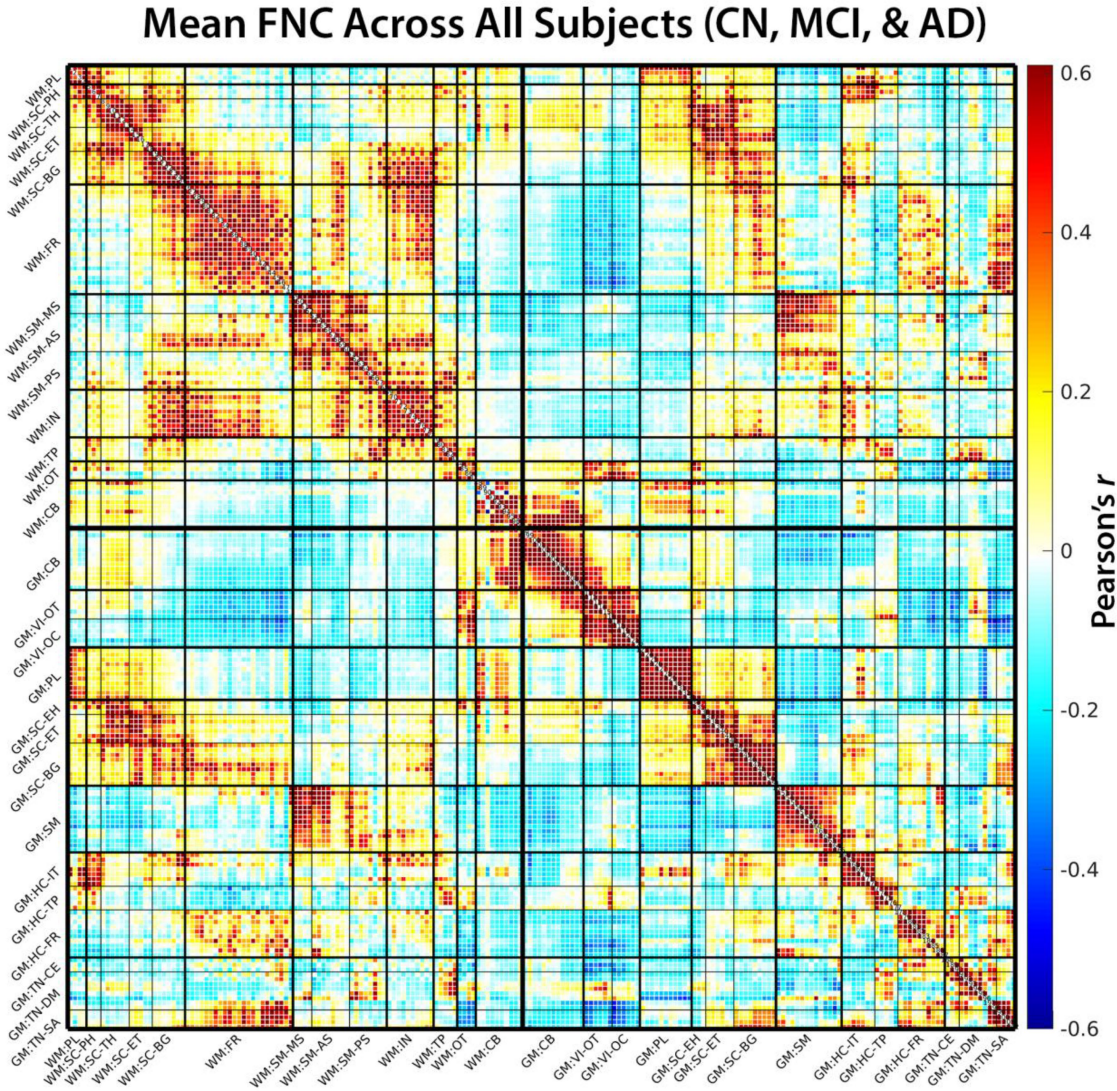
Mean FNC matrix of all subjects. The matrix displays mean functional network connectivity (FNC) for all subjects, calculated from a Pearson correlation between all intrinsic connectivity networks (ICNs). The heatmap ranges from *r* = −0.6 to 0.6, with negative values in blue and positive values in red. ICNs within the FNC matrix are grouped by their domain-subdomain labels, consistent with the NeuroMark 2.2 white matter (WM) and gray matter (GM) templates, indicated with a “WM:” or “GM:” prefix, respectively. The WM ICNs are grouped into 8 domains and 13 subdomains as follows: paralimbic (PL), subcortical-posterior hippocampal (SC-PH), subcortical-thalamic hippocampal (SC-TH), subcortical-extended thalamic (SC-ET), subcortical-basal ganglia (SC-BG), frontal (FR), sensorimotor-middle sensorimotor (SM-MS), sensorimotor-anterior sensorimotor (SM-AS), sensorimotor-posterior sensorimotor (SM-PS), insular (IN), temporoparietal (TP), occipitotemporal (OT), and cerebellar-brainstem (CB). The GM ICNs are grouped into 7 domains and 14 subdomains as follows: cerebellar (CB), visual-occipitotemporal (VI-OT), visual-occipital (VI-OC), paralimbic (PL), subcortical-extended hippocampal (SC-EH), subcortical-extended thalamic (SC-ET), subcortical-basal ganglia (SC-BG), sensorimotor (SM), higher cognition-insular-temporal (HC-IT), higher cognition-temporoparietal (HC-TP), higher cognition-frontal (HC-FR), triple network-central executive (TN-CE), triple network-default mode (TN-DM), and triple network-salience (TN-SA). The group-average FNC shows clear block organization in both WM and GM subdomains. WM subdomains form comparatively broader modules, while GM exhibits finer-grained modular structure. Notable WM–GM coupling is evident, particularly involving subcortical, frontal, and somatosensory subdomains.

FNC matrices showed a broadly similar large-scale organization across CN, MCI, and AD groups (Figure 2). In all three diagnostic categories, the combined WM and GM network representation exhibited a clear block structure corresponding to WM–WM, GM–GM, and WM–GM connections, as well as visually apparent block structure within sensorimotor, visual, cerebellar, frontal, and subcortical systems. This preserved global architecture suggests that large-scale network organization remains broadly similar across the AD spectrum. This preserved global architecture indicates that core network modules remain identifiable across the AD spectrum, despite substantial reconfiguration of specific within- and cross-tissue connections described below.

### Widespread white matter and cross-tissue connectivity disruption in AD

Group comparisons across the AD spectrum revealed widespread alterations in functional connectivity involving both WM and GM networks. These disruptions were particularly evident in contrasts involving the AD group, AD versus CN and AD versus MCI, highlighting the involvement of WM and WM–GM interactions in disease progression.

**Figure 3:**
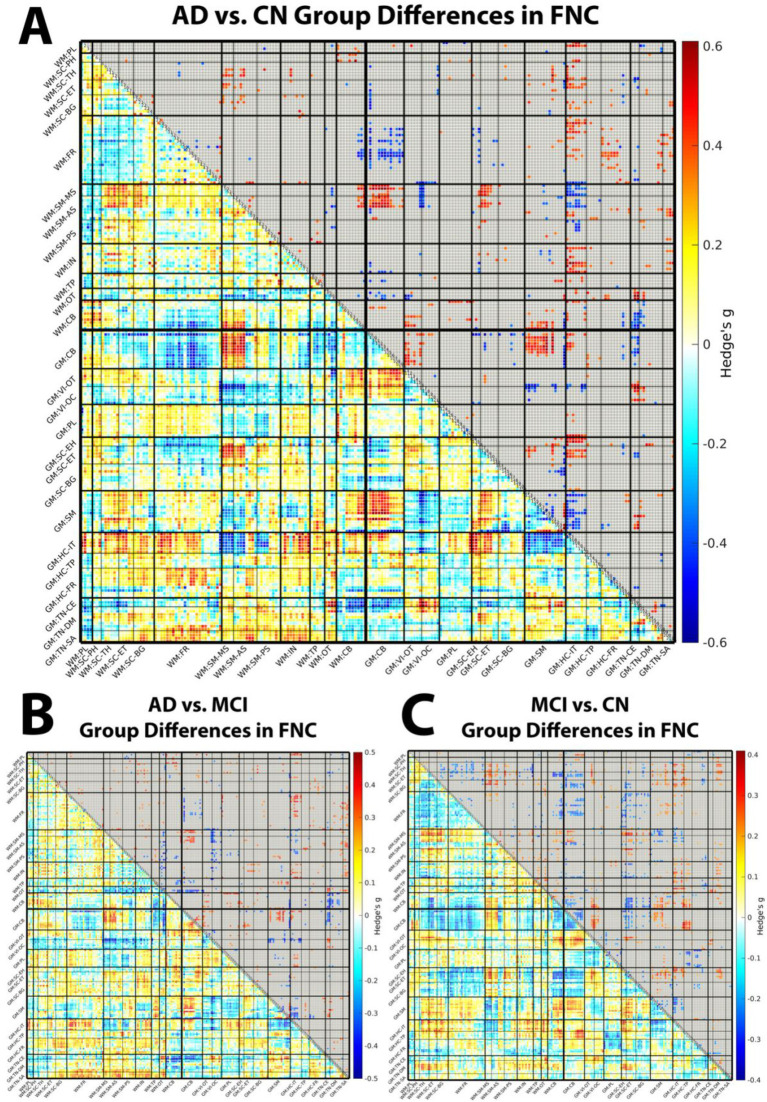
Group differences in functional network connectivity (FNC) across diagnostic stages. Difference matrices for **(A)** Alzheimer’s disease (AD) vs. cognitively normal (CN), **(B)** AD vs. mild cognitive impairment (MCI), and **(C)** MCI vs. CN contrasts. Each matrix summarizes edge-wise group differences in FNC between 202 ICNs, with the 97 white matter (WM) ICNs grouped into 8 domains and 13 subdomains as follows: paralimbic (PL), subcortical-posterior hippocampal (SC-PH), subcortical-thalamic hippocampal (SC-TH), subcortical-extended thalamic (SC-ET), subcortical-basal ganglia (SC-BG), frontal (FR), sensorimotor-middle sensorimotor (SM-MS), sensorimotor-anterior sensorimotor (SM-AS), sensorimotor-posterior sensorimotor (SM-PS), insular (IN), temporoparietal (TP), occipitotemporal (OT), and cerebellar-brainstem (CB). The 105 gray matter (GM) ICNs are grouped into 7 domains and 14 subdomains as follows: cerebellar (CB), visual-occipitotemporal (VI-OT), visual-occipital (VI-OC), paralimbic (PL), subcortical-extended hippocampal (SC-EH), subcortical-extended thalamic (SC-ET), subcortical-basal ganglia (SC-BG), sensorimotor (SM), higher cognition-insular-temporal (HC-IT), higher cognition-temporoparietal (HC-TP), higher cognition-frontal (HC-FR), triple network-central executive (TN-CE), triple network-default mode (TN-DM), and triple network-salience (TN-SA). Color values represent Hedges’ *g* effect sizes derived from two-sample *t*-tests comparing group differences in FNC after regressing out age, sex, and mean framewise displacement. Positive (red) values indicate higher connectivity in the first group (e.g., AD) relative to the second (e.g., CN), whereas negative (blue) values indicate lower connectivity. Edges are thresholded in the upper triangle, with non-significant group differences (false discovery rate–corrected *q* > 0.05) grayed out. Together, these matrices highlight widespread disruptions in WM–WM, GM–GM, and WM–GM connectivity, including prominent posterior WM hypoconnectivity, subcortical–sensorimotor reorganization, and early cerebellar/subcortical–frontal WM–GM dysconnectivity across the Alzheimer’s disease spectrum.

In the AD versus CN comparison (Figure 3A), we observed distinct alterations in WM–WM connectivity. Hypoconnectivity emerged within the occipitotemporal (OT) WM subdomain and in its connections to other WM networks, suggesting reduced integration across posterior WM pathways. In contrast, hyperconnectivity was evident between subcortical (SC) and sensorimotor (SM) WM domains, potentially reflecting altered subcortical–sensorimotor communication in AD.

Within the GM–GM domain, significant hypoconnectivity was observed between SM and hippocampal (HC) regions, implicating disrupted interactions between motor-related and memory-related systems. At the same time, enhanced connectivity between cerebellar (CB) and visual (VI) subdomains suggested atypical reorganization within visuomotor circuits.

Critically, several WM–GM interactions were also disrupted in AD relative to CN. These included reduced connectivity between GM–CB and WM–frontal (FR) networks, alongside increased coupling between GM–HC and WM–FR regions. Together, these patterns highlight altered communication between memory-related GM regions, cerebellar systems, and frontal WM pathways, indicating that AD-related network disruption is not confined to GM but extends prominently to cross-tissue interactions.

### Progressive reorganization of white matter–gray matter connectivity from MCI to AD

In the AD versus MCI comparison (Figure 3B), differential connectivity patterns continued to emerge, indicating progressive network reorganization beyond the MCI stage. At the WM–GM level, we observed increased connectivity between GM temporal and semantic subdomains, such as temporal pole and superior temporal sulcus regions, and WM–FR and WM–insular (IN) networks. These findings suggest that temporal and semantic GM systems become more tightly coupled to frontal and insular WM pathways as disease severity increases.

Concurrently, several GM–GM and WM–GM connections showed reduced connectivity in AD relative to MCI. Decreases were evident between GM–SM and GM–HC/insular–temporal (HC–IT) regions, as well as between GM–VI and WM–SM networks. WM–WM integration was also reduced, particularly between OT and SM domains, pointing to ongoing disconnection of posterior and sensorimotor WM pathways even between adjacent clinical stages. This combination of strengthened temporal–frontal/insular cross-tissue coupling and weakened sensorimotor and posterior pathways is consistent with a progressive, spatially nonuniform reorganization of both within-tissue and cross-tissue networks from MCI to AD.

### Early white matter–gray matter dysconnectivity in mild cognitive impairment

The MCI versus CN contrast (Figure 3C) revealed that WM–GM dysconnectivity is already present at the prodromal stage. The MCI cohort’s heterogeneity, including individuals who may remain stable versus those who convert to dementia, likely contributes to the observed variability in connectivity patterns. Nevertheless, the presence of significant WM–GM dysconnectivity in MCI relative to CN, combined with progressive reorganization from MCI to AD, supports WM functional connectivity as both an early marker of prodromal pathology and a dynamic indicator of disease progression across the AD continuum. Widespread reductions in WM–GM connectivity were most prominent between GM–CB and GM–SC regions and WM–FR subdomains, suggesting that cerebellar and subcortical influences on frontal WM pathways are diminished before the onset of overt dementia. Within the GM–GM domain, we observed reduced CB–CB connectivity accompanied by increased CB–VI coupling. This opposing pattern, loss of intra-cerebellar connectivity with heightened cerebellar–visual interactions, may reflect altered network organization in the face of emerging pathology.

Collectively, these results emphasize the evolving nature of WM involvement in AD. Altered connectivity within and across WM and GM domains from CN to MCI to AD supports the view that WM plays an active, dynamic role in the neurodegenerative process. These functional disruptions extend beyond structural degeneration alone and highlight the potential of WM-based functional connectivity measures as potential biomarkers for early diagnosis and disease monitoring. Across contrasts, cerebellar/subcortical–frontal WM–GM connectivity appears to be altered earliest (MCI vs. CN), with subsequent disruption of posterior OT and sensorimotor WM–WM connectivity and broader WM–GM reweighting in AD.

## Discussion

This study provides evidence that WM functional connectivity is altered across the AD spectrum and that these alterations are not restricted to GM networks. By jointly modeling WM and GM ICNs, we identified stage-dependent disruptions in FNC within WM, within GM, and across WM–GM connections in CN, MCI, and AD groups.

A consistent pattern across contrasts was reduced connectivity in posterior WM pathways, particularly within occipitotemporal (OT) subdomains, together with increased connectivity between subcortical (SC) and sensorimotor (SM) WM networks. The posterior hypoconnectivity is compatible with prior diffusion MRI reports of microstructural degradation in posterior association and visual tracts in AD (Mito et al., 2018) and suggests weakened functional coordination in these pathways. In contrast, SC–SM hyperconnectivity may reflect altered subcortical–sensorimotor coupling (Zhao et al., 2019).

At the GM level, we observed decreased connectivity between sensorimotor (SM) and hippocampal (HC) subdomains, indicating decoupling between memory-related and sensorimotor systems, in line with known hippocampal and default-mode disruptions in AD (Zhao et al., 2008). In addition, altered cerebellar (CB) connectivity, including reduced CB–CB coupling and increased CB–visual (VI) interactions, suggests a shift from intra-cerebellar coordination toward cerebellar–cortical and cerebellar–visual engagement. Prior work has implicated cerebellar contributions to visuomotor, navigational, and cognitive functions in aging and dementia (Jacobs et al., 2018), and the observed pattern is consistent with a redistribution of functional load across cerebellar and cortical systems.

Cross-tissue analyses indicated that WM–GM connections are affected early and evolve with disease severity. In AD, reduced connectivity between GM–CB/SC regions and WM–frontal (FR) networks, together with increased connectivity between GM–HC and WM–FR, indicates abnormal reweighting of inputs to frontal executive pathways from cerebellar, subcortical, and hippocampal systems. These findings generalize earlier GM-only reports of disrupted fronto-hippocampal coupling in AD (Wang et al., 2006; Li et al., 2021) by showing that part of this disruption is expressed in WM–GM interfaces. Importantly, WM–GM dysconnectivity was already present in MCI relative to CN, especially in cerebellar/subcortical–frontal connections, and continued to change between MCI and AD. This pattern is consistent with the broader literature describing coexisting hypo- and hyperconnectivity and network instability in AD (Jones et al., 2016; Dai et al., 2019).

These results support the view that WM functional connectivity provides information beyond structural metrics alone. Structural imaging robustly demonstrates WM degradation in AD (Mito et al., 2018), but does not quantify how communication across distributed systems is functionally altered. An important question is whether observed functional changes are secondary to structural degeneration or represent independent mechanisms. Our posterior WM hypoconnectivity aligns with diffusion MRI reports of microstructural degradation, suggesting functional changes may partially reflect axonal and myelin loss, while hyperconnectivity patterns cannot be easily explained by structural degradation alone and may reflect compensatory reorganization or maladaptive reconfiguration. By estimating FNC from WM and GM ICNs, we capture how the system-level organization of WM and WM–GM interactions changes with disease. This aligns with work showing that WM exhibits reliable, low-frequency BOLD fluctuations that are temporally coherent and spatially organized (Peer et al., 2017; Gore et al., 2019; Itkyal et al., 2025), and extends it by demonstrating that these fluctuations carry disease-relevant, stage-specific information. The template-guided ICA framework offers validated methodological advantages over traditional approaches: the WM ICN template was derived from over 100,000 fMRI scans, ensuring reproducibility and generalizability, and unlike atlas-based methods, it adapts to individual scans while maintaining intersubject correspondence (Itkyal et al., 2025). Using this framework, 97 stable WM ICNs were identified with high reproducibility (spatial correlation > 0.8 across model orders), providing a robust foundation for characterizing WM functional connectivity alterations. Importantly, the present study represents an early application of the NeuroMark WM template–guided ICA framework to investigate functional connectivity across the Alzheimer’s disease spectrum. By enabling data-driven identification of WM functional networks while preserving cross-subject correspondence, this approach moves beyond fixed ROI-based methods and provides a unified framework for systematically examining WM–WM, GM–GM, and WM–GM connectivity, offering a scalable and reproducible strategy for studying whole-brain functional organization in health and disease.

Several limitations should be noted. First, the present analysis is restricted to static FNC; dynamic FNC methods may reveal time-varying connectivity states that are more sensitive to early or subtle abnormalities. Second, although the use of validated WM and GM ICN templates and a guided ICA framework improves reproducibility, template-based approaches remain sensitive to residual noise, individual anatomical variability, and potential template–subject mismatch. Third, the cross-sectional design and lack of behavioral, cognitive, or molecular (e.g., amyloid, tau) measures limit inference regarding temporal trajectories, clinical correlates, and mechanistic interpretation of the observed connectivity changes. Finally, multi-site acquisition in ADNI introduces scanner and site heterogeneity that may not be fully accounted for despite standard preprocessing and covariate adjustment. Future work integrating longitudinal data and cognitive measures will be important to distinguish compensatory from maladaptive reorganization.

The WM template, derived from over 100,000 scans and validated on independent datasets, guides data-driven ICA estimation of subject-specific WM networks, allowing reproducible analysis of WM–WM, GM–GM, and WM–GM connectivity while adapting to individual anatomy and avoiding fixed ROI assumptions, with detailed spatial map analysis in AD and MCI left for future work. Future work should also examine WM and WM–GM connectivity using dynamic FNC approaches, integrate diffusion MRI to relate structural and functional alterations within specific tracts, and incorporate cognitive and biomarker measures in longitudinal designs to test whether WM-related connectivity metrics predict conversion or rate of decline. Task-based fMRI could further clarify how WM–GM connectivity differences modulate performance in memory, visuospatial, or motor tasks. In addition, a direct comparison of structural connectivity in WM with these WM FNC results may yield additional information about how brain structure and brain function are jointly and differentially impacted by neurodegenerative disease (Majeed et al., 2011; LaGrow et al., 2026; Bolt et al., 2022; Liu et al., 2018).

In summary, this study demonstrates that WM functional network connectivity can be robustly quantified from resting-state fMRI and is systematically altered across the AD spectrum (CN to MCI to AD). Joint analysis of WM and GM networks reveals widespread disruptions in intra- and cross-tissue connectivity, including WM–GM interactions that emerge early and progress with disease severity. These findings support the integration of WM functional measures into connectivity frameworks for AD and highlight their potential utility as biomarkers for diagnosis, stratification, and disease monitoring.

## Data Availability

The datasets presented in this study can be found in online repositories. The names of the repository/repositories and accession number(s) can be found in the article/Supplementary material.

## Footnotes

1
https://trendscenter.org/software/gift/

## Supplementary material

SUPPLEMENTARY FIGURE 1 Workflow for template-guided spatially constrained ICA and post-processing of WM–GM functional connectivity. The numbered boxes indicate the main inputs and analytical steps: (1) derivation of the Neuromark 2.2 gray matter template from 900 ICs across more than 100,000 fMRI scans, (2) use of a reproducible white matter ICN template, (3) analysis of 789 ADNI resting-state fMRI scans, (4) preprocessing, (5–6) application of gray matter and white matter network templates, (7) subject-level decomposition with GIFT spatially constrained ICA to obtain spatial maps and time courses, (8) computation of functional network connectivity matrices, and (9) covariate-adjusted group testing.
