# Supplementary figures and images for: Investigating white matter functional network connectivity across the Alzheimer’s disease spectrum using resting-state fMRI
Source: Front Neuroimaging. 2026 Apr 23;5:1796824. doi: 10.3389/fnimg.2026.1796824 (PMC13149195; doi:10.3389/fnimg.2026.1796824)

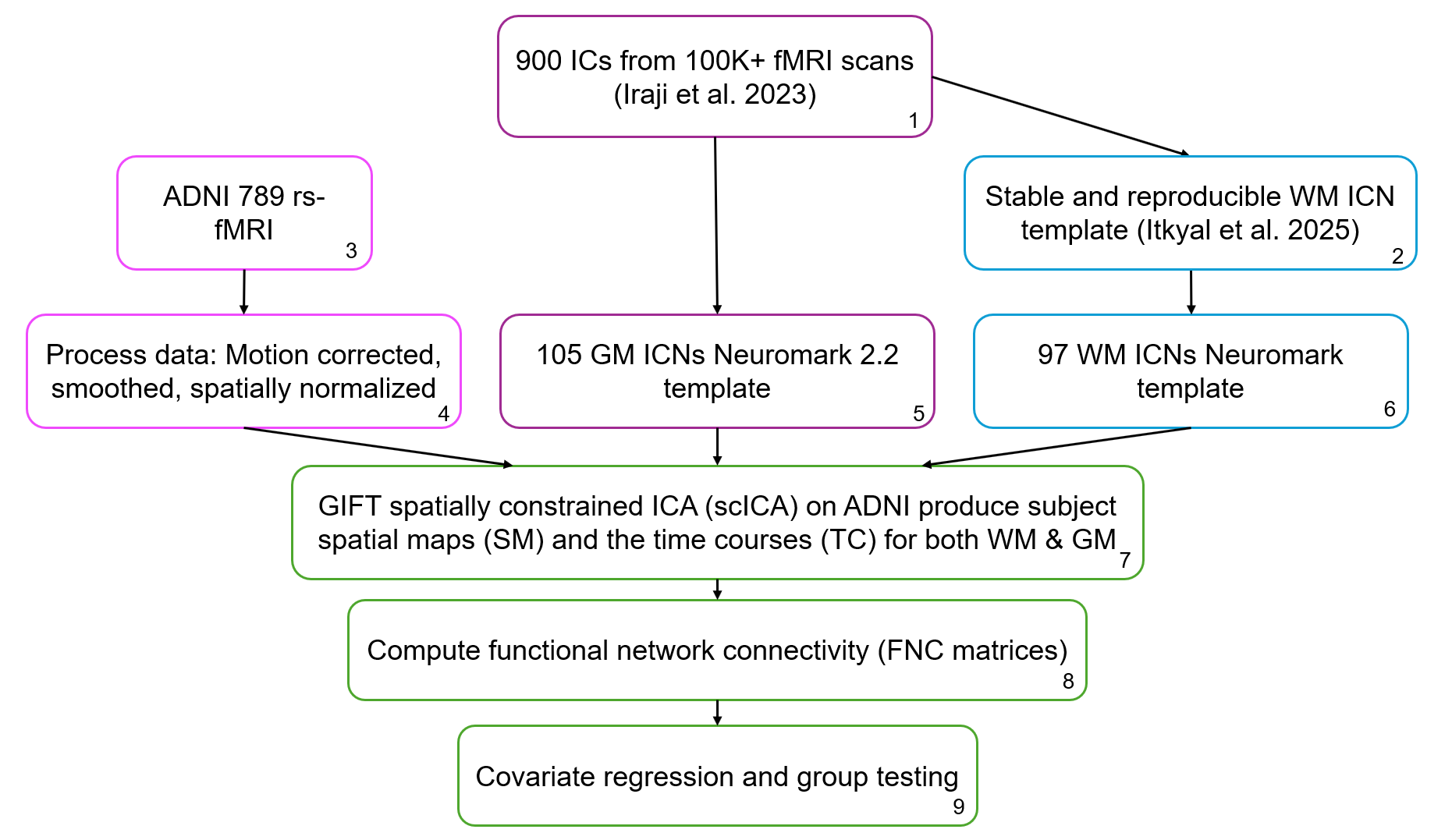

Supplement: SUPPLEMENTARY FIGURE 1 — Workflow for template-guided spatially constrained ICA and post-processing of WM–GM functional connectivity. The numbered boxes indicate the main inputs and analytical steps: (1) derivation of the Neuromark 2.2 gray matter template from 900 ICs across more than 100,000 fMRI scans, (2) use of a reproducible white matter ICN template, (3) analysis of 789 ADNI resting-state fMRI scans, (4) preprocessing, (5–6) application of gray matter and white matter network templates, (7) subject-level decomposition with GIFT spatially constrained ICA to obtain spatial maps and time courses, (8) computation of functional network connectivity matrices, and (9) covariate-adjusted group testing. [file Image_1.png]
